# Chondroma of the Bladder: An Atypical Localization

**DOI:** 10.1155/2017/6548314

**Published:** 2017-08-24

**Authors:** M. Tamayo-Jover, A. Nazco-Deroy, R. González-Álvarez, H. Álvarez-Argüelles Cabrera, B. Padilla-Fernández, T. Concepción-Masip

**Affiliations:** ^1^Urology Department, General Hospital of La Palma, Breña Alta, Spain; ^2^Pathology Department, University Hospital of Canary Islands, San Cristóbal de La Laguna, Spain; ^3^Urology Department, University Hospital of Canary Islands, San Cristóbal de La Laguna, Spain

## Abstract

Chondroma is a benign tumour of mesenchymal origin that is composed of cartilage and rarely located in soft tissues, being described so far only in four cases, as located in the bladder, according to our knowledge. We describe the fifth case of a 67-year-old woman who consulted for microscopic haematuria, with an endoscopic finding of submucosal nodular image in the anterior wall of the bladder, which after resection and the histologic study shows cartilage and fibroconnective tissue, in part hyalinised, and positive immunohistochemical staining of cells with vimentin and S-100; this fact can support the diagnosis of bladder chondroma.

## 1. Case Report

A 67-year-old woman with a history of hypertension, dyslipidemia, hypothyroidism, osteoporosis, and mixed anxiety-depressive disorder, as well as a history of mastectomy plus axillary lymphadenectomy for breast cancer 16 years ago and in remission, is referred to the Urology Department for persistent microscopic haematuria.

The patient denied having any abdominal pain, dysuria, frequency, tenesmus, or some other urinary symptoms. The urinalysis confirms the presence of persistent microscopic haematuria of between 60 and 100 red blood cells per field, and a urine culture is performed which comes back negative. Urologic ultrasound was requested, which showed the presence of an avascular echogenic image with a polypoid aspect of approximately 16 × 20 mm in the anterior wall of the bladder, so that endoscopic examination is performed under anaesthesia, evidencing raising or elevation of the bladder mucosa, with a cystic appearance on the anterior wall of the bladder of approximately 15 mm, which is completely resected with a resection loop for subsequent pathological analysis. The recovery of the patient was uneventful.

**Figure 1:**
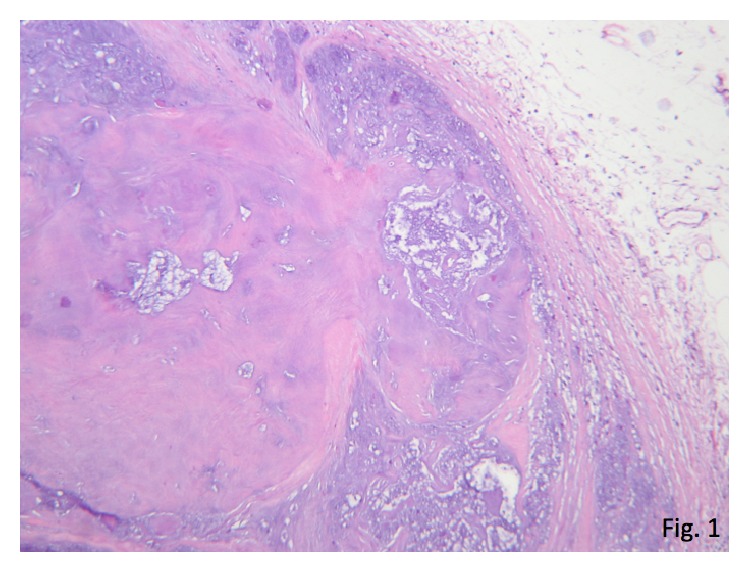
Well delimited and lobulated formation, consisting of cartilaginous appearance tissue (H-E, 50x).

**Figure 2:**
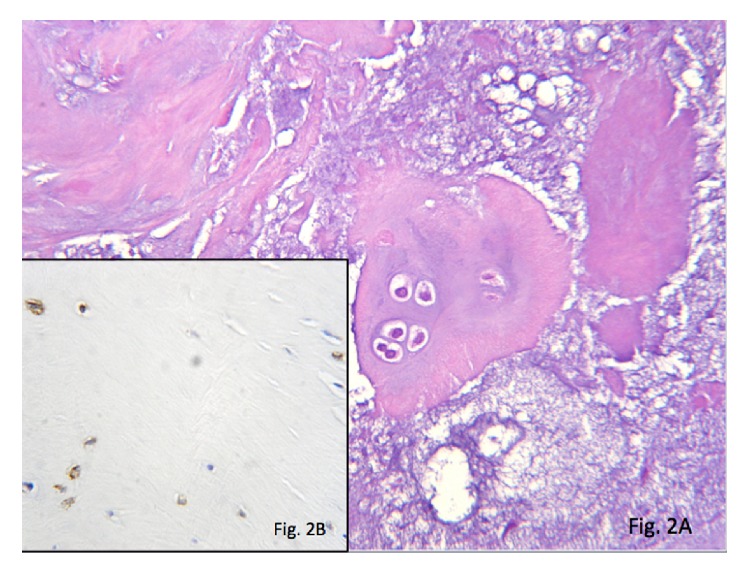
(A) In the tumour, mature chondrocyte nests are found in a basophilic chondroid matrix with myxoid loose areas. (B) In the lower left corner of the image, positive immunostaining for S-100 is seen in the cytoplasm and nucleus of chondrocyte-like cells (200x).

Macroscopically, two white-brown fragments of bladder wall with muscular layer were analysed. The fragments together measure 14 × 7 × 4 mm. Microscopically, one of the fragments corresponds to fibroconnective tissue compressed by nodular and lobulated tumour composed of hyaline cartilaginous, without cellular atypia and with basophilic areas (Figures 1 and 2(A)).

Immunohistochemical study of the tumoural tissue was immunostain positive for S-100 (Figure 2(B)) and vimentin and negative for CK, p53, and Ki 67, which supports the diagnosis of chondroma of the bladder mucosa.

In two years of follow-up, the patient remained asymptomatic, with negative urine cultures, normal cystoscopy with previous resection scar on the anterior wall without pathological findings, and urologic ultrasound within normal limits.

## 2. Discussion

Chondroma is a benign tumour of mesenchymal origin and slow growth that is composed of cartilage and is rarely located in soft tissues, being more commonly described in the fingers, limbs, and head or neck [1]. To date, only four cases of this type of tumour have been described in the bladder according to our knowledge [2–5], which makes it an extremely rare pathologic condition.

Our case, as in the previous four, has been in women with an age between the fifth and seventh decade of life. Three of the previously described patients presented some type of abdominal pain at the time of diagnosis, whereas, in the present case, it was an incidental finding during the study of microscopic haematuria, very similar to the last case described in the literature. The endoscopic image seems to coincide in the 5 cases, since it is a smooth submucosal tumour and located in the anterior bladder wall.

Soft tissue chondromas usually affect both sexes equally, between 40 and 70 years of age [6]. The origin of this type of tumour is not clear; however, Huggins (1931) described the ability of the urothelium to induce bone and cartilaginous metaplasia [7].

Some studies seem to correlate the pathogenesis of this tumour with the possible genetic influence demonstrated in monosomies, trisomies, translocations, and rearrangement of trisomy 11 in cytogenetic studies of soft tissue chondromas [8]; however, clonal mutation has not yet been demonstrated [9].

From the histopathological point of view, the findings can be variable and may exist from mature forms with hyaline cartilage arranged in lobes (some with fibrosis, ossification, or myxoid areas) to immature forms with preponderance of chondroblasts [10]. Like the previous ones, our case corresponds to mature cartilage form.

The main differential diagnosis to be taken into account is chondrosarcoma, which presents a malignant course and can mean a great challenge for the pathologist to be able to differentiate a low-grade chondrosarcoma from a soft tissue chondroma [2, 3].

Therefore, soft tissue chondroma in the bladder is a possible pathology, although rare, and every pathologist should think of it as a possible entity in cases of masses with presence of subepithelial hyaline tissue.

## 3. Conclusions

It is necessary to accumulate more cases to obtain adequate conclusions and to better understand the causes of this pathology; however we can say that bladder chondroma is presumably an extremely rare condition and benign behaviour and must be taken into account during the pathological analysis in order to reach its possible diagnosis.

## References

[1] Nayler S., Heim S., Fletcher C. D. M., Unni K. K., Mertens F. (2002). Soft tissue chondroma. *Pathology and Genetics of Tumours of Soft Tissue and Bone*.

[2] Pauwels C. F., Van Den Broecke C., Demeyer J. M., De Potter C. R. (1998). Chondroma of the bladder. *Virchows Archiv*.

[3] Perrino C. M., Pohar K. S., Zynger D. L. (2012). Urinary bladder chondroma. *Virchows Archiv*.

[4] Carter M. D., Rendon R. A., Merrimen J. (2015). A rare case of bladder condroma. *Canadian Urological Association Journal*.

[5] Tazeh N. N., Scott K., Damodaran S., Huang W., Downs T. M. (2017). Chondroma of the bladder: a case report and review of the literature. *Urology*.

[6] Kishore S., Thakur B., Azad S., Kudesia S. (2016). Scrotal chondroma in a young male: a rare case report. *Indian Journal of Pathology and Microbiology*.

[7] Huggins C. B. (1931). The formation of bone under the influence of epithelium of the urinary tract. *Archives of Surgery*.

[8] Cin P. D., Qi H., Sciot R., Van Den Berghe H. (1997). Involvement of chromosomes 6 and 11 in a soft tissue chondroma. *Cancer Genetics and Cytogenetics*.

[9] Mandahl N., Heim S., Arheden K., Rydholm A., Willén H., Mitelman F. (1990). Chromosomal rearrangements in chondromatous tumors. *Cancer*.

[10] Enzinger F. M., Weiss S. W. (1977). *Cartilaginous Tumors and Tumorlike Lesions of Soft Tissue*.

